# Extracorporeal membrane oxygenation in trauma patients: a systematic review

**DOI:** 10.1186/s13017-020-00331-2

**Published:** 2020-09-11

**Authors:** Changtian Wang, Lei Zhang, Tao Qin, Zhilong Xi, Lei Sun, Haiwei Wu, Demin Li

**Affiliations:** Department of Cardiovascular Surgery, School Medicine, Jinling Hospital, Nanjing University, Nanjing, People’s Republic of China

**Keywords:** Trauma, Extracorporeal membrane oxygenation (ECMO), Systematic review

## Abstract

**Background:**

Extracorporeal membrane oxygenation (ECMO) has evolved considerably over the past two decades and has been gradually utilized in severe trauma. However, the indications for the use of ECMO in trauma remain uncertain and the clinical outcomes are different. We performed a systematic review to provide an overall estimate of the current performance of ECMO in the treatment of trauma patients.

**Materials and methods:**

We searched PubMed and MEDLINE databases up to the end of December 2019 for studies on ECMO in trauma. The PRISMA statement was followed. Data on demographics of the patient, mechanism of injury, injury severity scores (ISS), details of ECMO strategies, and clinical outcome were extracted.

**Results:**

A total of 58 articles (19 retrospective reports and 39 case reports) were deemed eligible and included. In total, 548 patients received ECMO treatment for severe trauma (adult 517; children 31; mean age of adults 34.9 ± 12.3 years). Blunt trauma (85.4%) was the primary injury mechanism, and 128 patients had traumatic brain injury (TBI). The mean ISS was 38.1 ± 15.0. A total of 71.3% of patients were initially treated with VV ECMO, and 24.5% were placed on VA ECMO. The median time on ECMO was 9.6 days, and the median time to ECMO was 5.7 days. A total of 60% of patients received initially heparin anticoagulation. Bleeding (22.9%) and thrombosis (19%) were the most common complications. Ischemia of the lower extremities occurred in 9 patients. The overall hospital mortality was 30.3%.

**Conclusions:**

ECMO has been gradually utilized in a lifesaving capacity in severe trauma patients, and the feasibility and advantages of this technique are becoming widely accepted. The safety and effectiveness of ECMO in trauma require further study. Several problems with ECMO in trauma, including the role of VA-ECMO, the time to institute ECMO, and the anticoagulation strategy remain controversial and must be solved in future studies.

## Background

Polytrauma is a leading cause of death among adults [1]. The major causes of early death are hemorrhagic shock, hypoxemia, hypothermia, metabolic acidosis, coagulopathy, and severe traumatic brain injury. Among these causes, hypoxemia or acute respiratory distress syndrome (ARDS) still represents an important and frequent contributing factor toward morbidity and mortality after trauma [2, 3]. A recent systematic review demonstrated no change in the mortality of trauma-induced ARDS over the last several decades, and the mortality ranges from 20.6 to 25.8% [4].

Extracorporeal membrane oxygenation (ECMO) is a simplified version of the heart-lung machine that can restore adequate tissue perfusion and oxygenation, achieve quick rewarming, and infuse massive fluids or blood products. Over the past two decades, ECMO has evolved considerably, especially in ECMO devices (i.e., ECMO devices, centrifugal pump techniques, complete heparin-coated circuits, more efficient oxygenators, or device miniaturization). Since the first successful use of ECMO in adults for acute posttraumatic respiratory failure reported by Hill et al. in 1972 [5], ECMO has advanced significantly and has become a salvage therapy that may provide an alternative form of management for cardiopulmonary failure in traumatic patients when conventional treatments have failed. However, some concerns remain uncertain in trauma patients, such as the time to ECMO and anticoagulation, and the current clinical outcomes are different. Recently, survival rates for trauma patients undergoing ECMO have been shown to range from 44% to as high as 74.1% [6]. The aim of this systematic review is to provide an overall estimate of the current performance of ECMO in the treatment of trauma patients.

## Methods

This systematic review was performed and reported in line with the Preferred Reporting Items for Systematic Reviews and Meta-Analyses (PRISMA) statement [7]. We searched the PubMed and MEDLINE databases up to the end of December 2019 using medical subject headings and text words supplemented by scanning the bibliographies of the recovered articles. We combined “extracorporeal membrane oxygenation”, “ECMO”, “extracorporeal life support”, “ECLS”, “extracorporeal cardiopulmonary life support”, “trauma”, “multitrauma”, “polytrauma”, and “injury” using the Boolean operator “AND”. The results were limited to articles written in English. Two separate researchers (C.W. and L.Z.) analyzed the data set to ensure accuracy and to identify all available studies. The details of each publication were checked to avoid duplicates. Any differences were resolved by consensus.

### Inclusion criteria

All publications, including case reports and case series, reported the application of ECMO during the treatment of trauma patients. Only papers published in English that reported age, sex, mechanism of injury, injury severity score (ISS), details of ECMO, and clinical outcome were included.

### Exclusion criteria

Studies or cases reporting on ECMO for treatment as a bridge for delayed surgery, as support during an emergency operation, on rewarming in the treatment of hypothermia and on burns were not included in this review. Similarly, correspondence, expert opinions, and reviews were excluded. Cases that were not described in the details of demographic data, especially the details of ECMO and outcome, were also not included.

### Data extraction and risk of bias assessment

The following basic parameters were extracted from every publication: first author, date of publication and study, study design (e.g., retrospective study vs. case report), number of patients included, patient demographics, mechanism of injury, type of trauma, ISS, Glasgow coma scale (GCS), details of ECMO (time to ECMO, duration of ECMO, type of ECMO, access route of ECMO), anticoagulation management, mortality, cause of death and ECMO-related complications.

The risk of bias was assessed at the study level using Cochrane’s Collaboration Tool [8]. Through six domains, this tool evaluates the risk of bias and categorizes each study as high risk, low risk, or unclear risk.

### Statistical analysis

Data collected were organized on an Apple Numbers (version 6.6.2) spreadsheet. Descriptive statistics were used to describe demographic and continuous data (e.g., mean ± SD). Dichotomous variables are expressed as numbers with percentages.

## Results

**Fig. 1:**
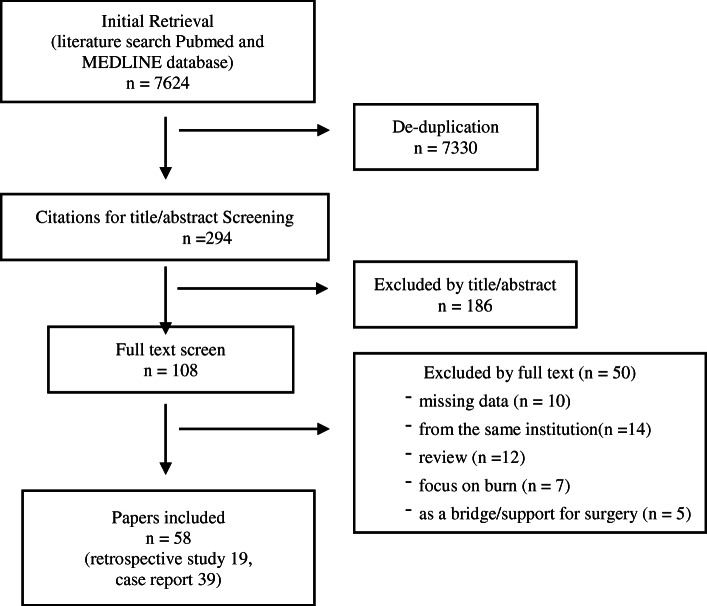
Flow diagram to illustrate the identification, selection, and exclusion of articles used in the review

The literature search yielded 7624 publications in the PubMed and MEDLINE databases. We screened them by title/abstract and full text. Finally, 58 publications (19 retrospective reports and 39 case reports) that focused on ECMO in trauma were identified from the literature and included in the analysis, spanning a period of time ranging from 1972 to 2019 (Fig. 1). All studies were retrospective studies or case series, with no prospective controlled studies.

### Demographic data

A total of 548 trauma patients requiring ECMO were described, including 517 adults (94.3%) and 31 children (5.7%). There was a wide difference in sample size ranging from 1 patient to 85 patients. The retrospective reports had 497 patients, and case reports included 51 patients. The majority of patients were male (*n* = 441, 80.1%), and 99 were female (18.1%). One report did not provide the patient’s gender. In adults, the mean age was 34.9 ± 12.3 years (range 18–86 years), and the youngest patient in the group of children was only 21 months old.

Four reports did not provide data on the cause of injury (*n* = 175). In the remaining 373 cases, traffic accidents (*n* = 254, 68.1%) were the most common cause of injury, followed by falling (*n* = 38, 10.2%). Gunshots (*n* = 13, 3.5%) and stabbing (*n* = 6, 1.6%) were the major causes of penetrating trauma. The data on the mechanism of trauma were available in 53 papers (*n* = 384). Blunt trauma (*n* = 328, 85.4%) was the primary injury mechanism with very high injury severity and a significant thoracic component. In this review, 259 (*n* = 67.4%) patients had chest trauma. Penetration occurred in sixteen patients (4.2%). A total of 128 patients had traumatic brain injury (TBI) in 52 publications, including 404 trauma patients. Twenty-four papers provided the Injury Severity Score (ISS). The mean ISS was 38.1 ± 15.0 (range 4–75). The mean Glasgow Coma Scale (GCS) was 7.3 ± 4.7 (range 3–15) in 19 papers.

The overall hospital mortality was 30.3% (*n* = 166). There was a wide variation in mortality rates among the studies (0–71.4%). In the 19 retrospective reports, the overall mortality was 33% (*n* = 164), and in case reports, it was 3.9% (*n* = 2). Six deaths occurred in the pediatric group (19.4%). The leading cause of death was MOF (*n* = 34). The following causes were sepsis (*n* = 23), cardiorespiratory failure (*n* = 22), cerebrovascular accident (*n* = 18), death after withdrawal of care (*n* = 11), massive hemorrhage (*n* = 10), ECLS failure (*n* = 4), and mesenteric ischemia (*n* = 1). The cause of death was unknown or missing among 68 patients (Table 1).

**Table 1:** Case series summary of ECMO in trauma

Author	Study types	Year of publish/study	Size	Gender(M/F)	Age(year, range)	Causes of trauma	Mechanism of trauma	TBI	ISS	GCS	Hospital mortality*n* (%)	Cause of death
Kruit N et al [9]	Retrospective study	2019(2011–2017)	52	42/10	33 (18-72)	Traffic accident 36 Falling 8 Assault 4 Helicopter crash 1 Speedboat crash 1 Missing data 2	–	19 (3 died)	35 (6–75)	–	6 (11.5%)	Cerebral herniation 2 Mesenteric ischemia 1 Massive hemorrhage 1 Unknown 2
Fenton SJ et al [10]	Retrospective study	2019(2009–2016)	6	4/2	4.8 (3–8)	Traffic accident 5 Crush injury 1	Blunt chest trauma	4	36 (27–40)	3	1 (16.7%)	Large right-sided ischemic cerebrovascular accident
Lang NW et al [11]	Retrospective study	2019(2002**–**2016)	18	11/7	29.5 (1-64)	Traffic Accident 10 Falling 4 Stab 2 Other 2	Blunt polytrauma	5	34.5 (16**–**50)	6.6 ± 4.8(3–15)	12 (66.6%)	MOF, sepsis, hypoxic cerebral edema
Menaker J et al [12]	Retrospective study	2018(2015**–**2016)	18	15/3	28.5 (24-43)	blunt mechanism 12 missing data 6	–	–	27 (21–41)	–	4 (22%)	Shock and coagulopathy 3, primary neurologic injury 1
Wu MY et al [13]	Retrospective study	2018(2006–2014)	36	31/5	36 (27–49)	Traffic accident 24 Falling 5 Burn 3 Stab 1 Electrocution 1 Near-drowning 1 Compressing injury 1	–	4 (3 died)	29 (19–45)	–	15 (41.7%)	-
Strumwasser A et al [6]	Retrospective study	2018(2016–2017)	7	7/0	41 ± 15 ( 23–63 )	Traffic accident 5 Gunshot 2	Blunt polytrauma 5Open trauma 2	0	32.5 ± 14.1 (16–54)	–	5 (71.4%)	Multisystem organ failure and “comfort measures only"
Ull C et al [14]	Retrospective study	2017(2008**–**2014)	49	44/5	49.9 (16.6**–**86.2)	**–**	**–**	**–**	32 (4**–**66)	**–**	17 (34.7%)	-
Kim HS et al [15]	Retrospective study	2017(2007–2015)	9	8/1	48.0 (20.5–62.0)	Traffic accident 4 Gunshot 1 Crush injury 2 Falling 2	–	–	–	–	1 (11.1%)	–
Ahmad SB et al [16]	Retrospective study	2017(2006–2015)	39	29/10	Mean 28 Survivors 35 (25–45) Nonsurvivors 27 ( 22–42)	Traffic accident 26 Falling 3 Gunshot 6 Stab 1 Impalement 1 Near-drowning 1 Crush injury 1	Blunt polytrauma 31 Penetrating trauma 8	11	Mean 29 Survivors 25 (18–32) Nonsurvivors 41 (26–50)	–	22 (56%)	Cardiorespiratory arrest 10 Brain death 1 Died after withdrawal of care 11
Chen TH et al [17]	Retrospective study	2016(2009**–**2012)	7	6/1	31 (21**–**49)	Traffic Accident 5 Falling 2	Blunt chest trauma	4	36 (27**–**57)	**–**	3 (43.8%)	Irreversible brain damage with vasodilatory shock; Septic shock with MOF
Bosarge PL et al [18]	Retrospective study	2016(2013–2014)	15	15/0	36 (25–47)	–	Severe TBI	2	26.0 (17.0–34.0)	–	2 (13.3%)	Hypoxia
Jacobs JV et al [19]	Retrospective study	2015(1998–2014)	85	71/14	28.9 ± 1.1	–	Blunt chest trauma	14	–	–	22 (25.9%)	–
Guirand DM et al [20]	Retrospective study	2014(2001–2009)	26	20/6	33.0 (16–55 )	–	Blunt trauma 21	–	29.0 (12.4)	–	11 (42%)	–
Bonacchi M et al [21]	Retrospective study	2013(2008–2012)	18	12/6	46.3 ± 17.6 (15-69)	Traffic Accident 15 Falling 2 Crash 1	Blunt polytrauma	7	53 ± 17 (18–75)	–	13 (72.2%)	ECMO failure 4 septic MOF 2 cerebral death 7
Biderman P et al [22]	Retrospective study	2013(2006–2011)	10	6/4	29.8 ± 7.7 (19–42)	Traffic accident 6 Falling 2 Bombing 1 pedestrian injury 1	Blunt polytrauma 9 Cardiac perforation 1	7 (1 died)	50.3 ± 10.5(29–57)	–	3 (30%)	Septic 2 Cardiogenic shock 1
Ried M et al [23]	Retrospective study	2013(2002–2012)	52	49/3	32 ± 14 (16–72)	Traffic accident 38 Blast injury 9 Falling 4 Blunt trauma 1	Blunt chest trauma 40	30	58.9 ± 10.5	–	11 (21%)	Multiorgan failure 9 Fulminant bleeding 1 Cerebral hypoxia and bleeding 1
Cordell-Smith JA et al [24]	retrospective study	2006(1992–2002)	28	24/4	27 (18–49)	Traffic accident 24 Sporting accidents 2 Extensive crush 2	Blunt polytrauma	–	18	–	8 (28.6%)	Irreversible cardiogenic failure 4 Sepsis 4
Fortenberry JD et al [25]	Retrospective study	2003(1991–2001)	8	NA	12.5 (21 months—29 years)Pediatric 4 (21 months—19 years)	Traffic accident 7 Gunshot 1	Blunt polytrauma 7 Penetrating 1	2	–	–	2 (25%)Pediatric1, adult 1	Pulmonary fat emboli and necrotic lung tissue 1 Severe pulmonary hypertension 1
Senunas LE et al [26]	Retrospective study	1997(1988–1994)	14	4/10	19 (5–47)	Traffic accident 14	Orthopaedic trauma (severe pulmonary compromise)	-	19 (9-29)	14	6 (42.9% Children 4 Adult 2)	Bleeding 5ARF 1
Robba C et al [27]	Case report	2017 (2000–2016)	4	4/0	31,58,36,54	Traffic accident 2 Falling 2	Blunt trauma	2	25,41,50,50	15, 6, 15, 3	1 (25%)	Malignant intracranial hypertension
Amos T et al [28]	Case report	2019	1	1/0	21	Traffic accident	Thoracic compartment syndrome	0	–	3	0	–
Matsumoto S et al [29]	Case report	2019	1	0/1	64	Traffic accident	Blunt chest trauma	0	–	–	0	–
Puzio TJ et al [30]	Case report	2019	1	1/0	2.5	Dog bite injury	Severe head, neck, and facial injuries	1	–	3	0	–
Ogawa F et al [31]	Case report	2019	1	1/0	32	Traffic accident	Blunt chest trauma	0	33	–	0	–
Lorini FL et al [32]	Case report	2019	1	1/0	21	Hit by rolled rock	Blunt chest trauma	0	–	–	0	–
Wang FY et al [33]	Case report	2019	1	1/0	47	Compressing	Blunt chest trauma	0	–	–	0	–
Moon SH et al [34]	Case report	2018	1	0/1	26	Traffic accident	Blunt chest trauma	0	–	–	0	–
Chico Carballas JI et al [35]	Case report	2018	1	1/0	26	Traffic accident	Brain trauma	1	25	4	0	–
Yuan KC et al [36]	Case report	2008	2	2/0	18,38	Traffic crash	Blunt chest trauma	1	–	–	0	**–**
Yoann L et al [37]	Case report	2013	1	0/1	37	Horse riding accident	Blunt chest trauma with cardiac rupture	0	–	–	0	–
Louro J et al [38]	Case report	2018	1	1/0	14	Falling	Blunt polytrauma	0	–	15	0	–
Tu Y et al [39]	Case report	2018	1	1/0	24	Traffic accident	Blunt polytrauma	0	–	–	0	–
Lee N et al [40]	Case report	2015	1	1/0	28	Stab	Penetrating cardiac trauma	0	–	–	0	–
Stroehle M et al [41]	Case report	2017	1	1/0	17	Hit and run over by a railroad engine	Blunt polytrauma	1	59	3	0	–
Park JM et al [42]	Case report	2014	1	0/1	40	Traffic accident	Blunt polytrauma	1	–	7	0	–
Skarda D et al [43]	Case report	2012	3	2/1	17,8,15	Traffic accident 2 Gunshot 1	Blunt polytraum 2 Perforation trauma 1	2	–	3, 14, 15	0	–
Yamada T et al [44]	Case report	2014	1	1/0	56	Falling	Blunt chest trauma	0	–	8	0	–
Firstenberg MS et al [45]	Case report	2012	1	1/0	27	Traffic accident	Blunt chest trauma	1	–	–	0	–
Ballouhey Q et al [46]	Case report	2013	1	0/1	32-month	Traffic accident	Blunt chest trauma	0	–	–	0	–
Campione A et al [47]	Case report	2007	1	1/0	14	Go-cart race	Blunt chest trauma	0	–	–	0	–
Issa N et al [48]	Case report	2011	1	1/0	30	Traffic accident	Fractures, splenic injury,cardiorespiratory arrest	0	–	–	0	–
Rosenthal A et al [49]	Case report	2009	1	1/0	42	Gunshot	Open chest trauma	0	–	–	0	–
Tunney R et al [50]	Case report	2018	1	1/0	19	Traffic accident	Blunt polytrauma	0	–	–	0	–
Chauhan A et al [51]	Case report	2014	1	0/1	18	Traffic accident	Blunt polytrauma	0	–	–	0	–
Bassi E et al [52]	Case report	2011	1	1/0	48	Traffic accident	Blunt polytrauma	1	–	3	1(100%)	extensive brain infarction
Filippini S et al [53]	Case report	2013	1	1/0	25	Traffic accident	Blunt polytrauma	0	–	–	0	–
Wen PH et al [54]	Case report	2015	1	1/0	19	Traffic accident	Blunt polytrauma	0	–	8	0	–
Madershahian N et al [55]	Case report	2007	3	2/1	19,48,26	Traffic accident 2 Falling 1	Blunt polytrauma	1	–	4, 12, 4	0	–
Lee SK et al [56]	Case report	2017	1	1/0	21	Traffic accident	Blunt polytrauma	0	–	–	0	–
Biscotti M et al [57]	Case report	2015	2	2/0	18, 20	Traffic accident	TBI	2	27, 33	14, 3	0	–
Lee OJ et al [58]	Case report	2019	1	1/0	10	Traffic accident	Blunt polytrauma	0	25	–	0	–
Messing JA et al [59]	Case report	2014	1	1/0	21	Traffic accident	Blunt polytrauma	1	38	–	0	–
Voelckel W et al [60]	Case report	1998	2	2/0	29,12	Traffic accident hit by a falling tree	Blunt chest trauma	0	–	–	0	–
Yen TS et al [61]	Case report	2008	1	1/0	21	Traffic accident	TBI	1	-	12	0	–
Muellenbach RM et al [62]	Case report	2012	3	3/0	53, 16, 28	Traffic accident Falling Traffic accident	Blunt polytrauma	3	59, 66, 66	4, 3, 6	0	–
Valette X et al [63]	Case report	2011	1	1/0	17	Traffic accident	Blunt chest trauma	0	–	–	0	–
Barreda E et al [64]	Case report	2007	1	1/0	28	Gunshot	Penetrating chest trauma	0	–	15	0	–
Hill JD et al [5]	Case report	1972	1	1/0	24	Traffic accident	Blunt polytrauma	0	–	–	0	–
Total	Retrospective study 19case Report 39	1972–2019	548	M 441F 99	adult 34.9 ± 12.3(18–86)Child (21 months-18)	Traffic accident 254 Falling 38 Gunshot 13 Stabbing 6	Blunt 328 Penetrating 16 Chest trauma 259	128	38.1 ± 15.0(4–75)	7.3 ± 4.7 (3–15)	166 (30.3%)adult (30.9%) child (19.4%)	

Notes: Values are expressed as numbers and % or mean±SD.

acute renal failure, *ECMO* Extracorporeal membrane oxygenation, *GCS* Glasgow Coma Scale, *ISS* Injury Severity Score, *MOF* Multiple organ failure, *TBI* Traumatic brain injury

### ECMO strategies

A total of 391 patients (71.3%) were initially treated with VV ECMO, and 134 patients (24.5%) were initially placed on VA ECMO. Twelve of those treated with VV ECMO converted to VA ECMO support due to persistent hemodynamic instability. Only two cases on VA ECMO were converted to VV ECMO.

A total of 377 patients (68.8%) were cannulated peripherally, three patients were centrally cannulated (femoral vein-aortic root; right atrium-aorta; right atrium-pulmonary artery), and 168 patients (30.7%) did not describe the type of cannulation. Single dual-lumen catheters were used in this group, but the details could not be identified from the literature.

The median time on ECMO was 9.6 days in this group, and the longest was 114 days. The duration before ECMO support was recorded differently, including from injury to ECMO, from ER to ECMO, from the onset of ARDS to ECMO, and from the ventilator to ECMO. The median time before ECMO support was 5.7 days. In 28 cases, this time was recorded approximately (<1 day) (Table 2).

**Table 2:** The details of ECMO support

ECMO support	Size (***n***, %)
VV-ECMO—no. (%)	391 (71.4)
VA-ECMO—no. (%)	134 (24.5)
VV convert to VA—no. (%)	12 (2.2)
VA convert to VV—no. (%)	2 (0.4)
missing type—no. (%)	23 (4.2)
PC-mode—no. (%)	377 (68.8)
CC-mode—no. (%)	3 (0.5)
Missing mode—no. (%)	168 (30.7)
Duration of ECMO (days, mean)	9.6
Time to ECMO (days, mean)	5.7^a^

Notes: Values are expressed as numbers and % or mean±SD.

*CC* Central cannulation, *ECMO* Extracorporeal membrane oxygenation, *PC* Peripheral cannulation, *VA-ECMO* Veno-arterial extracorporeal membrane oxygenation, *VV-ECMO* Veno-venous extracorporeal membrane oxygenation

^a^The record of time to ECMO was different in publications, including from ER to ECMO, from the onset of ARDS to ECMO, from the ventilator to ECMO

### Anticoagulation

The anticoagulation strategy on ECMO in trauma patients differed among institutions. Seven papers included 164 cases (30%) that did not provide details of anticoagulation. A total of 329 patients (60%) received anticoagulation while on ECMO, and 55 patients did not receive anticoagulation. In the anticoagulation group, 135 (24.6%) patients received heparin systemic anticoagulation. Heparin titration (heparin-minimized strategy) was applied to 80 (14.6%) patients. A total of 101 (18.4%) patients were initially managed with heparin (did not provide the later strategy), while 13 (2.4%) patients were initially managed with heparin (1–3 days); then, heparin was stopped due to bleeding or other causes. In the anticoagulation-free group, 16 (2.9%) patients did not receive any anticoagulation while on ECMO. Initial heparin-free therapy occurred in 14 (2.6%) patients, and 24 (4.4%) patients received initial heparin-free therapy (2.5 h—5 days) followed by heparin systemic anticoagulation. Nafamostat mesilate was reported on ECMO in two studies, but the details could not be extracted.

Activated clotting time (ACT) and activated partial thromboplastin time (aPTT) were the two most common coagulation parameters, which were used in 124 cases and 120 cases, respectively. ACT between 180 and 220 s was the most targeted time. The target aPTT was between 40 and 80 s. In one study, the target was a TEG-reactive (R) time of two times the nonheparinized baseline.

### Complications

**Fig. 2:**
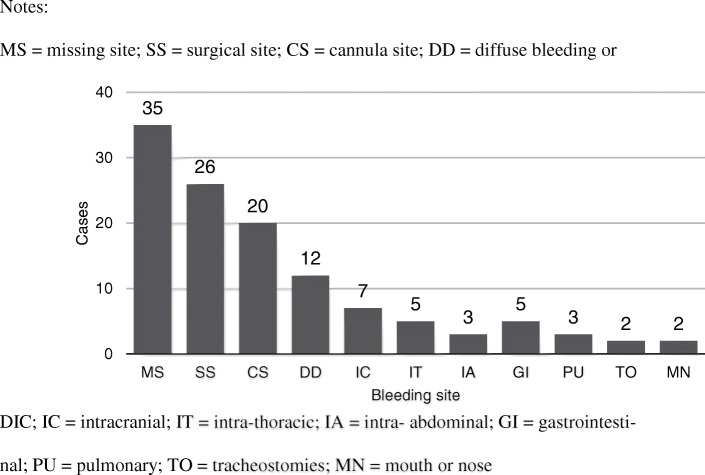
The summary of documented bleeding complication during ECMO in trauma. Notes: MS = missing site; SS = surgical site; CS = cannula site; DD = diffuse bleeding or DIC; IC = intracranial; IT = intra-thoracic; IA = intra- abdominal; GI = gastrointestinal; PU = pulmonary; TO = tracheostomies; MN = mouth or nose

A total of 104 patients (22.9%) had documented bleeding complications at 120 sites, including surgical site bleeding (*n* = 26), cannula site bleeding (*n* = 20), diffuse bleeding or DIC (*n* = 12), intracranial bleeding (*n* = 7), intrathoracic bleeding (*n* = 5), intra-abdominal bleeding (*n* = 3), gastrointestinal bleeding (*n* = 5), pulmonary bleeding (*n* = 3), bleeding from tracheostomies (*n* = 2), and bleeding from the mouth or nose (*n* = 2). Thirty-five sites were missing (Fig. 2). Four papers, which included 94 patients, did not report bleeding complications. The surgical site was the most common bleeding site, followed by the cannula site. The incidence of bleeding complications was different in series reports, ranging from 0 to 87.5%, and the majority of these events did not require surgical intervention.

**Fig. 3:**
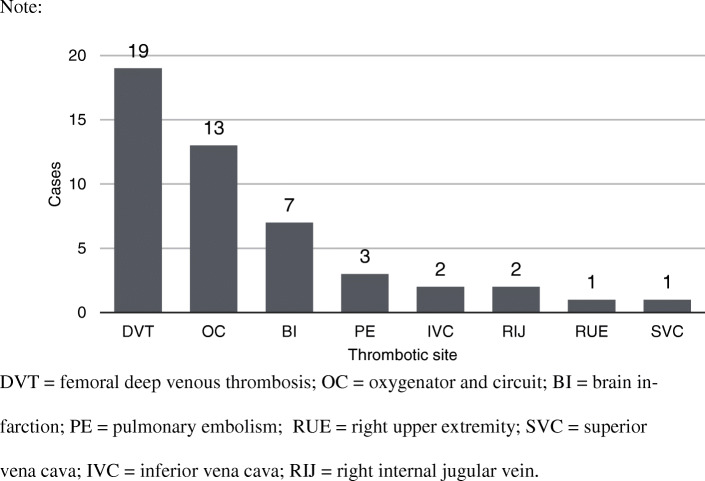
The summary of documented thrombotic complication during ECMO in trauma. Notes: DVT = femoral deep venous thrombosis; OC = oxygenator and circuit; BI = brain infarction; PE = pulmonary embolism; RUE = right upper extremity; SVC = superior vena cava; IVC = inferior vena cava; RIJ = right internal jugular vein

Thrombotic complications are listed in Fig. 3, including femoral deep venous thrombosis (DVT), oxygenator and circuit clotting, brain infarction, pulmonary embolism, inferior vena cava (IVC) thrombus, right internal jugular vein (RIJ) thrombus, right upper extremity (RUE) thrombus, and superior vena cava (SVC) thrombus. Nine papers (296 cases) did not document complications. Forty-eight of 252 patients (19%) experienced thrombotic complications. A total of 39.6% of patients had femoral DVT. The incidence of thrombi in oxygenators and circuits was 27.1%.

The other ECMO-related complications occurred in 25 cases reported in 15 publications, including ischemia of the lower extremity, abdominal compartment syndrome, brain swelling, acute lung edema, acute pancreatitis, accidental removal of a cannula, pseudoaneurysm developed on the site of the cannula, and secondary sclerosing cholangitis in critically ill patients (SSC-CIP) (Table 3). Ischemia of the lower extremities (*n* = 9) was the most common cannula-related complication, and 3 patients underwent fasciotomy. The other complications were very rare.

**Table 3:** The summary of other ECMO-related complications in trauma

Complications	Size
Ischemia of lower extremity (fasciotomy 3)	9
Abdominal compartment syndrome	2
Brain swelling	2
Acute lung edema	1
Acute pancreatitis	1
Accidental removal of a cannula	1
Pseudoaneurysm developed on the site of cannula	1
SSC-CIP	1
Non-record	7
Total	25

Notes: *ECMO* Extracorporeal membrane oxygenation, *SSC-CIP* Secondary sclerosing cholangitis in critically ill patients

## Discussion

Generally, the lung is the first organ to fail after severe trauma (failure after 3.7 ± 2.8 days) [65]. In our review, ECMO has been used successfully in a series of trauma patients, but the evidence of benefit is still limited. From our investigation, the reported mortality in publications ranged from 11.1 to 71.4%, with the exception of case reports. The mean hospital mortality was 30.3%, while in the pediatric group, it was 19.4%. Mortality remains high, and the cause should take into account the overall injury as well as those that are ECMO-related, because most of the population in this study was young and had a median age of 34.9 years, with good cardiopulmonary function at baseline. In a multicenter study by Kruit et al. [9], fifty-two patients who experienced trauma and were supported with ECMO were identified from the five ECMO centers. The overall hospital mortality was 11.5%, and the overall 180-day mortality was 15%. These preliminary results suggest that ECMO may provide survival benefits and seems to be safe in selected trauma populations. However, owing to the difference of injury, indications and time of ECMO, and follow-up data, the benefits of ECMO in trauma should be confirmed through further studies, for example, randomized control trials.

When to initiate ECMO is a challenge to clinicians. In this study, the time to initiate ECMO in the trauma patients was different, ranging from 0 to 24 days, and the mean time was 5.7 days. Kruit et al. demonstrated that there was no significant difference between survivors and nonsurvivors in time from injury to ECMO commencement [9]. Ahmad et al. [16] also did not find an association between the time to initiation of ECMO and mortality. Summary data suggest that delaying the institution of ECMO may cause irreversible pulmonary and cardiac injuries in addition to other organs and lead to poor outcomes [66]. Bosarge et al. evaluated a series of 15 patients with severe ARDS who underwent early ECMO (mean 1.9 days) and found that the patients in the early ECMO group had improved survival (64%) compared with historical controls (13%), suggesting that ECMO should be considered at the early onset of severe ARDS to improve survival [18]. Early ECMO initiation may offer advantages such as reducing ventilator time to prevent iatrogenic lung damage from high-pressure and high FiO2 ventilation, supplying adequate oxygenation and/or tissue perfusion, and providing lung and heart rest. However, ECMO initiation may increase the bleeding risk in the early stages of trauma due to heparin systemic anticoagulation, especially in patients with intracranial or active systemic bleeding.

VV ECMO with peripheral cannulation (PC) is the most common model of ECMO in trauma. In this review, 71.4% of patients received VV ECMO support, and 24.5% received VA ECMO. The study based on the ELSO registry [19] showed a trend toward the use of venovenous ECMO compared with venoarterial ECMO, as 79.4% of survivors were treated with venovenous ECMO compared with 59.1% of nonsurvivors. One recent systematic review combining 215 trauma patients supported with ECMO reported a survival-to-discharge rate ranging from 56 to 89% after VV ECMO and 42 to 63% after VA ECMO [3]. In the study reported by Lang et al. [11], VA ECMO was applied in 15 cases, and 11 patients (73%) died. However, limited studies have examined outcomes for VA ECMO in trauma patients. VA ECMO is indicated for cardiopulmonary support rather than for pulmonary support alone, which enables the restricted and congested heart to recover by unloading the heart. Severe chest trauma in many cases can cause acute lethal cardiac failure primarily by contusion of the heart or secondary by pulmonary contusion. These patients might benefit from ECMO. A recent study [13] using ECMO to treat patients with advanced shock or respiratory failure showed different mortality rates between VA and VV ECMO: 64.3% in VA ECMO and 27.3% in VV ECMO. It is likely that patients requiring VA ECMO had poorer survival rates because they required hemodynamic support and not simply because VA ECMO was used. Therefore, much work still needs to be done to determine the effects and safety of VA ECMO in severe trauma patients.

Therapeutic heparin potentially increases the risk of bleeding, especially in polytrauma with TBI and/or intracranial bleeding. In this review, there are different anticoagulation strategies in the literature, including full heparin systemic anticoagulation, a heparin-minimized strategy, and all heparin-free, initially heparin-free and delayed heparin-free treatments, and there was a wide range (1.9–87.5%) of reported bleeding complications. According to the Extracorporeal Life Support Organization Registry Report 2012 [67], the main complication during VV-ECMO is bleeding, which occurs in 17 to 21.3% of cases. In the report by Ahmad et al [16], the use of anticoagulation was significantly associated with survival: 94% of survivors were anticoagulated versus only 55% of nonsurvivors. However, several reports have shown that patients can be successfully managed on heparin-free ECMO without increasing the extent of the bleeding [17, 22, 28, 29, 35, 38]. These may benefit from the technological advancements in ECMO, including the use of more efficient membrane oxygenators, centrifugal pumps, miniaturization of circuits, and heparin-bonded circuitry, which have allowed ECMO use with little or no anticoagulation. Anticoagulation should be individually tailored, taking into account the severity of the trauma, the timing, and active bleeding.

In this literature review, 128 patients suffering from traumatic brain injury (TBI) were included. Unfortunately, the details of anticoagulation and mortality could not be identified. Biderman et al. [22] reported a series of 10 patients, including 7 with TBI and six patients who survived. The median GCS score on arrival was 7 (range 5–9) and improved in all survivors to 14 (range 12–15). All but two patients regained normal neurologic status during follow-up. Of the 10 patients, only 3 could receive heparin during the first 48 h. In those patients who could not receive heparin, high blood flow (4–5 L/min) was maintained to prevent clotting. In a multicenter study [9], 52 traumatic patients were included, and 19 had TBI. Twelve patients with TBI (63%) were anticoagulated, and 3 with TBI died (16%). In another series report [17] including 7 consecutive severe trauma patients and 4 with TBI, VV-ECLS was not withheld from patients with TBI despite evidence of intracranial bleeding. Four patients were successfully discharged, and three of these survivors had concomitant TBI without neurologic sequelae. TBI is not contraindicated for ECMO, and routine management, including mild hypothermia (34 °C), can be tolerated under heparin-free VV ECMO [17, 68]. The current findings do not support neurological injury as an absolute contraindication to ECMO.

### Limitations

This systematic review presents limitations. All of the included publications on ECMO technology in trauma still consist of either retrospective studies or case series with limited data or case reports, and thus, the present report possesses all the inherent limitations, including the weak of the evidence, geographical bias, publication bias, search bias, methodology bias. Another limitation is the number and quality of the studies available for qualitative analysis. Some studies had incomplete data. Finally, follow-up data and long-term survival data are not yet available from this analysis. Therefore, a general conclusion from a solid statistical analysis with adequate samples is lacking.

## Conclusions

Our systematic review illustrates that ECMO has been gradually utilized in a lifesaving capacity in patients with severe trauma, and the feasibility and advantages of this technique are becoming widely accepted. However, the safety and effectiveness of ECMO in trauma require further study. Several problems with ECMO in trauma, including the role of VA-ECMO, the time to institute ECMO, and the anticoagulation strategy remain controversial and must be solved in the future studies. Indeed, clinical randomized control trials with large samples and long-term survival data are needed.

## Data Availability

All data generated or analyzed during this study are included in this published article [and its supplementary information files].

## Abbreviations

ACT: Activated clotting time
ARDS: Acute respiratory distress syndrome
aPTT: Activated partial thromboplastin time
DIC: Disseminated intravascular coagulation
DVT: Deep venous thrombosis
ECLS: Extracorporeal cardiopulmonary life support
ECMO: Extracorporeal membrane oxygenation
GCS: Glasgow coma scale
ISS: Injury severity scores
IVC: Inferior vena cava
MOF: Multiple organ failure
PRISMA: The preferred reporting items for systematic reviews and meta-analyses
RIJ: Right internal jugular vein
RUE: Right upper extremity
SVC: Superior vena cava
TBI: Traumatic brain injury; thrombus
VA-ECMO: Veno-arterial extracorporeal membrane oxygenation
VV-ECMO: Veno-venous extracorporeal membrane oxygenation
